# Development and Evaluation of a Method for Automated Detection of Spreading Depolarizations in the Injured Human Brain

**DOI:** 10.1007/s12028-021-01228-x

**Published:** 2021-07-26

**Authors:** Sharon Jewell, Stephen Hobson, Grant Brewer, Michelle Rogers, Jed A. Hartings, Brandon Foreman, José-Pedro Lavrador, Michael Sole, Clemens Pahl, Martyn G. Boutelle, Anthony J. Strong

**Affiliations:** 1 Department of Bioengineering, Imperial College London, London, UK; 2 Department of Basic and Clinical Neuroscience, Institute of Psychiatry, Academic Neuroscience Centre, King’s College London, Room A1.27, De Crespigny Park, Box 41, London, SE5 8AF UK; 3 Cybula Ltd, York, UK; 4 Department of Neurosurgery, College of Medicine, University of Cincinnati, Cincinnati, OH USA; 5 Department of Neurology and Rehabilitation Medicine, College of Medicine, University of Cincinnati, Cincinnati, OH USA; 6 Department of Neurosurgery, King’s College Hospital, London, UK; 7 Department of Intensive Care Medicine, King’s College Hospital, London, UK

**Keywords:** Cortical spreading depression, Brain contusion, Subdural hematoma, Subarachnoid hemorrhage, Electroencephalography, Computing methodologies

## Abstract

**Background:**

Spreading depolarizations (SDs) occur in some 60% of patients receiving intensive care following severe traumatic brain injury and often occur at a higher incidence following serious subarachnoid hemorrhage and malignant hemisphere stroke (MHS); they are independently associated with worse clinical outcome. Detection of SDs to guide clinical management, as is now being advocated, currently requires continuous and skilled monitoring of the electrocorticogram (ECoG), frequently extending over many days.

**Methods:**

We developed and evaluated in two clinical intensive care units (ICU) a software routine capable of detecting SDs both in real time at the bedside and retrospectively and also capable of displaying patterns of their occurrence with time. We tested this prototype software in 91 data files, each of approximately 24 h, from 18 patients, and the results were compared with those of manual assessment (“ground truth”) by an experienced assessor blind to the software outputs.

**Results:**

The software successfully detected SDs in real time at the bedside, including in patients with clusters of SDs. Counts of SDs by software (dependent variable) were compared with ground truth by the investigator (independent) using linear regression. The slope of the regression was 0.7855 (95% confidence interval 0.7149–0.8561); a slope value of 1.0 lies outside the 95% confidence interval of the slope, representing significant undersensitivity of 79%. *R*^2^ was 0.8415.

**Conclusions:**

Despite significant undersensitivity, there was no additional loss of sensitivity at high SD counts, thus ensuring that dense clusters of depolarizations of particular pathogenic potential can be detected by software and depicted to clinicians in real time and also be archived.

**Supplementary Information:**

The online version contains supplementary material available at 10.1007/s12028-021-01228-x.

## Introduction

A core precept in the delivery of neurocritical care is to minimize the occurrence of secondary insults, such as hypoxia, arterial hypotension, and pyrexia. A novel class of secondary insult has emerged in recent years and is believed to contribute to expansion of a focal lesion resulting from ischemic or TBI and may prove to be a realistic target for therapy. Since their first unequivocal demonstration in patients with acute brain injury in 2002 [1], SDs^1^ have become recognized as a secondary insult, occurring in TBI [2], aneurysmal subarachnoid hemorrhage (aSAH) [3], MHS [4], and intracerebral hematoma [5].

Originally described by Leao [6], SD is a wave of mass depolarization of neurons and glia in cerebral gray matter leading to depression of the normal ECoG amplitude and is caused by massive transmembrane ionic shifts, which, if not quickly restored to their normal distributions, initiate a cascade leading to tissue necrosis. Evidence that some depolarizations can contribute to tissue necrosis and hence prejudice clinical outcome has been summarized in recent reviews, together with methods that have been developed for their detection in the injured human brain in the setting of the ICU [7–11]. Detailed work has established a relationship between adverse outcome from TBI and occurrence of spontaneous SDs [12, 13].

Two recent discussion articles have addressed the dilemmas implicit in designing a therapeutic approach when SDs are detected [14, 15]. As one potential therapy, the *N*-methyl-*D*-aspartate receptor antagonist ketamine has been the subject of pilot studies [16, 17] and is seen as a leading candidate for further assessment. Given the increasing adoption of SD monitoring and the trend toward intervention, a need emerges for fresh technical and analytical approaches to the detection and assessment of SDs that simplify the challenges of bedside monitoring and real-time interpretation. Simplified routines would facilitate clinical investigation and drug trials as well as inform hour-to-hour regular clinical management. Central to this is the ability for clinicians to visualize simultaneously and in simple summary form both SDs and the variables currently known to contribute to their occurrence. The principal goal of the work reported here was to provide intensivists without specialized experience of ECoG a software routine offering early warning of the likely presence of SDs, coupled with the option of reviewing the raw record of each candidate event. This is needed because very few clinical neurophysiology services have the technical and clinical experience to offer a real-time diagnostic service to neurocritical care colleagues, particularly one that is needed continuously and extends over several days. This deficiency is currently placing a limit on the ability of neurointensive care clinicians to adopt a method that they now appreciate would enable them to provide more personalized care that identifies and responds to specific recognized abnormalities potentially amenable to targeted treatment.

Closely allied to this is the need for an SD-detection algorithm to display its results within the context of changes in core pathophysiology that can assist in prioritizing changes in management. This report therefore describes the development and validation of a software routine designed to detect and archive, in real time, SDs in the ECoG data stream from a patient with acute brain injury receiving intensive care. The SD-detection software is accessed by the clinician within a wider software facility (Neuromonitor) designed to display SD occurrence in the context of the patient’s current physiology; we illustrate briefly how the combination of features is delivered but focus on assessing the performance of the SD-detection software.

## Methods

### Patient Recruitment

Consecutive electrophysiological records were obtained from patients aged 16 years or older of either sex presenting with acute brain injury requiring emergency craniotomy (TBI, aSAH, MHS, or intracerebral hematoma). Patients were included if they underwent ECoG monitoring for SDs via subdural linear strip electrodes placed at the conclusion of surgery and before wound closure. At the University of Cincinnati (UC), the use of subdural strip electrodes for monitoring was approved by the institutional review board as part of an ongoing prospective observational study protocol, and each patient’s legally authorized representative provided written informed consent. At King’s College Hospital (KCH), written consent was obtained from either the next of kin or a consultant/faculty neurosurgeon who was not an investigator, as approved by the research ethics committee. For those without an immediately available next of kin, the use of subdural strip electrodes for SD monitoring was explained at the first opportunity with an offer to terminate research procedures. In no case was consent declined.

### Surgery and Intensive Care

As in previous reports on this topic [18], a unilateral frontotemporal or bifrontal craniotomy was conducted in accordance with clinical indications, and any acute subdural hematoma was removed. Any intracerebral hematoma exerting a mass effect was evacuated, and hemostasis was secured. Prior to closure, a Wyler ECoG strip (six or eight platinum contacts, exposed Ø2.3 mm, 10 mm center to center; Ad-Tech, Oak Creek, WI) was placed on the brain surface on tissue judged to be viable (whether edematous or hyperemic) and typically 1–2 cm distant from the contusion resection margin. In a limited number of cases, an additional probe, typically a brain tissue oxygen pressure (P_ti_O_2_) sensor (Licox; Integra, Tullamore, Ireland; or Raumedic, Helmbrechts, Germany), was inserted into the parenchyma adjacent to the ECoG strip. No scalp electroencephalogram data were collected for this study. Following surgery, the patient was returned to the ICU, and continuous ECoG commenced. Neurocritical care practices followed written guidelines that were based on those published by the Brain Trauma Foundation (*Guidelines for the Management of Severe TBI, Fourth Edition*; September 2016) and had changed little from our previous joint article [18]. ECoG recordings were terminated, and electrode strips were removed at the bedside by gentle traction when invasive neuromonitoring was no longer clinically required. No hemorrhagic or infectious complications were associated with the electrode strip.

### Software Development History and Study Design

When funding was received (please see Source of support), a detailed specification was developed by intensivists, neurosurgeons, and bioengineers (London) for a suite of software to (1) extract high-resolution time series data from bedside monitors, together with continuous ECoG from a six-contact subdural electrode strip placed under direct vision, at the conclusion of emergency surgery and (2) continuously monitor the ECoG output and detect SDs. The developers of the SD detector algorithm based their code on criteria established by members of the Co-Operative Studies of Brain Injury Depolarizations collaboration between 2003 and 2010 and described in detail by Dreier et al. [10] (for details, please see below). The SD-detection algorithm was progressively refined in an iterative dialogue between developers and clinicians with access to ECoG data acquired in the period 2010–2014. Clinical summaries of the development data sets used are given in Table 1. The dialogue continued over the same period, punctuated by work on other functionality in the overall Neuromonitor software package. During this time, generation of the separately frequency-filtered ECoG data streams relied on the proprietary filtering software within LabChart (ADInstruments, Bella Vista, Australia).

**Table 1:** Development set: summary of demographics and surgery

Patient ID	Age (yr)	Gender	Lesion and surgery	Number of data sets analysed	Hours recorded	Total SDs
*Barcelona*						
1	51	M	MHS-L: Decompressive hemicraniectomy	15	157	34
2	44	F	MHS-L: Decompressive hemicraniectomy	7	144	0
3	37	M	TBI, (L acute SDH): Left Decompressive hemicraniectomy	3	69	1
4	50	M	MHS-L: Decompressive hemicraniectomy	10	182	3
5	54	M	MHS-L: Decompressive hemicraniectomy	3	23.4	29
6	64	M	MHS-R: Decompressive hemicraniectomy	4	50	9
*London*						
1	67	M	TBI ( fall): R acute SDH: craniotomy	2	53	23
2	44	F	TBI (Alcohol: fall): L temporal contusion: craniectomy	1	55	24
3	31	M	TBI (Alcohol: fall): R frontotemporal contusions: craniotomy	3	99	22
4	54	M	TBI (Fall): R frontotemporoparietal contusions: craniectomy	1	51	9
5	61	M	TBI (Alcohol: fall). R parietal EDH, SDH, ICH: craniotomy	5	101	7
6	39	M	TBI: (Alcohol and& seizures: fall),. L acute SDH: frontoparietal craniotomy	4	61	9
7	39	M	TBI (Fall): L frontotemporal acute SDH: craniectomy and contusion evacuation	3	65	3
8	79	F	TBI (Fall): R frontoparietal acute SDH: craniotomy	9	71	101
9	28	M	TBI (Alcohol and& drugs: fall): L frontotemporal contusion: craniotomy	3	50	0
10	73	M	TBI (Fall): EDH and bifrontal contusions L > R:. L craniotomy and frontal partial lobectomy	3	31	5
11	40	M	TBI (Fall): multiple fractures,; R acute SDH and contusions: R temporoparietal craniotomy	5	70	0
12	46	M	TBI (Fall): L acute SDH and severe edema. L craniectomy	3	61	10
13	60	M	TBI, Coagulopathy: Bilateral acute SDH: R frontotemporal craniectomy	5	88	50
14	42	M	TBI (Alcohol and& epilepsy): R acute SDH: craniotomy	4	69	11
15	30	M	TBI (Pedestrian TBI): R acute SDH: craniectomy	3	47	8
Total	–	–	–	Total datasets analysed: 96	Total hours: 1597	86%

Eighteen patients (86%) experienced SDs

EDH, extradural hematoma; F, female; ICH, intracerebral hematoma; ID, iIdentity; L, left; M, male; MHS, Malignant hemisphere stroke; R, right; RTA, Road traffic accident; SAH, subarachnoid hemorrhage; SDs, spreading depolarizations; SDH, subdural hematoma; TBI, Traumatic brain injury

In 2018, KCH acquired a commercial system (Moberg CNS-310 Component Neuromonitoring System; Moberg Solutions, Inc., Ambler, PA) so as to better present SD and ongoing bedside monitoring data to the clinical team. This system is designed to assemble (and archive) on a common time axis not only conventional high-resolution signals from bedside monitors but also multiple channels of ECoG and additional external signals, such as P_ti_O_2_ and microdialysate metabolite values when available. In consequence, a new software interface of this system with our prototype SD detector and screen presentation software (Neuromonitor) was required. Notably, considerable fresh work was necessary, with newly coded frequency filters intended to replicate the performance of the original set; the work involved a further iterative process based on consecutive case recruitment. Once the KCH users were satisfied with the reliability of the new package (early 2019), no further development took place, and the data presented here—the validation data set (Table 2)—were acquired with the current single version (1.2.15) of the detector software. Thus, there is no available training data set, as conventionally defined in the context of neural network studies. Together with collaborators in the UC, we (KCH) monitored over similar time periods since early 2019 a consecutive total series of 24 patients. This report addresses only the technical performance of the SD detector packaged within Neuromonitor version 1.2.15.

**Table 2:** Validation set: summary of demographics and surgery

Patient ID	Age (yr)	Gender	Lesion and surgery	Number of data sets analysed	Hours recorded	Total SDs
*Cincinnati*						
1	76	F	TBI (fall): R acute SDH: hemicraniectomy	2	37.8	69
2	71	M	TBI (fall): R acute SDH: hemicraniectomy	1	42.0	5
3	60	F	TBI (fall): R acute SDH: hemicraniectomy	2	68.9	9
4	72	M	TBI (fall): R SAH: hemicraniectomy	8	133	46
5	23	M	TBI (RTA): hemicraniectomy	5	110.8	119
*London*						
1	28	F	TBI (RTA): R acute SDH and ICH: hemicraniectomy	17	389.8	61
2	30	M	TBI (assault): Bifrontal and basal mixed contusions: bifrontotemporal craniectomy	3	63.0	9
3	54	M	R basal ganglia spontaneous ICH: mini-craniotomy and: later, hemicraniectomy	3	72.4	0
4	39	M	TBI (RTA): craniotomy for L acute SDH	5	110.0	39
5	63	F	MHS subsequent to tumour resection: R decompressive hemicraniectomy	4	62.6	0
6	59	M	TBI (presumed fall): craniotomy for L acute SDH	7	130.7	28
7	66	M	TBI (fall): craniotomy for R acute SDH	7	141.9	52
8	58	M	TBI (RTA): Bifrontotemporal contusions and SAH: R acute SDH: R hemicraniectomy	4	91.6	0
9	43	M	TBI (Fall): R acute SDH:; R craniotomy	1	29.4	0
10	29	F	TBI (RTA): bifrontal craniectomy	9	205.9	7
11	54	M	TBI (RTA): craniotomy for L acute SDH	7	140.0	14
12	52	M	TBI (Fall): R temporal contusion and SAH: craniectomy: re-evacuation	3	33.4	0
13	27	M	TBI (RTA): L acute SDH and diffuse injury: bifrontal craniectomy	3	52.2	0
Total	–	–	–	Total datasets analysed: 91	Total hours: 1915.4	(SDs in:-) 67%

Twelve (67%) of the 18 patients experienced SDs

F, female; ID, Identity; ICH, Intracerebral hematoma; L, left; M, male; MHS, Malignant hemisphere stroke; R, right; RTA, Road traffic accident; SAH, subarachnoid hemorrhage; SDs, spreading depolarizations; SDH, subdural hematoma; TBI, Traumatic brain injury

### Monitoring Technology (Bedside)

Continuous digitized signals for arterial and intracranial pressures, end-tidal CO_2_, body temperature, and heart rate were fed via a serial connection from Philips Intellivue monitors to a Moberg CNS-310 Component Neuromonitoring System (Moberg Solutions, Inc.). ECoG strips were connected to a Moberg CNS Advanced ICU EEG Amplifier (40 channels; input frequency response: DC to Nyquist) feeding to the same CNS-310 system and digitized at 256 or 512 Hz. A sintered Ag/AgCl scalp electrode contralateral to the injury (KCH) or subdermal platinum needle placed at ipsilateral mastoid (UC) served as a far-field common reference. An Ag/AgCl scalp electrode served as ground. Systemic variables and ECoG were logged and displayed continuously in real time on the same time axes and archived. In all cases at KCH and one case at UC, the same data were fed also in real time through a peer-to-peer ethernet connection to a laptop PC hosting Neuromonitor prototype version 1.2.15 (initially written by Cybula Ltd, York, UK, as commissioned by a Health Innovation Challenge Fund grant), the index system for this evaluation of its diagnostic accuracy. It was not edited throughout the conduct of this study.

### Screen Presentation of Neuromonitor Software to Users

To meet the clinicians’ specifications, a horizontally time-scrolled screen was established with 12 rows, 11 of which were allocated to continuously monitored variables grouped as indices of oxygenation/respiratory function (end-tidal CO_2_, P_ti_O_2_, and systemic oxygen saturation), perfusion (mean arterial pressure, cerebral perfusion pressure, and intracranial pressure), brain temperature, and brain metabolism (implemented when data from rapid sampling cerebral microdialysis are available). Each individual square block in each row represents a 15-min average for a given variable, and blocks are color rendered according to the value relative to clinician-defined target values, thus constituting one example of a heatmap. A white arrow is located within the square to indicate abnormally low or high departure from normal range. The screen width is 12 h.

### Software Detection of SD Events

The 12th row (electrophysiology) depicts the results from online software analysis of six channels of ECoG data. The detection algorithms were developed and evaluated by using a data set containing 64.6 days of data from 21 patients at two different hospitals (the development cohort; Table 1), with classifications compared with ground truth files scored manually by co-authors at KCH. There is no overlap between this earlier development data set or cohort and the validation data set (Table 2) used to calculate the results presented here.

The analysis takes a bottom-up approach, with low-level features identified in individual channels and then combined together and classified as either an artifact, CSD, ISD, or CSD/ISD, following the international consensus criteria for manual review and scoring [10, 19].

**Fig. 1:**
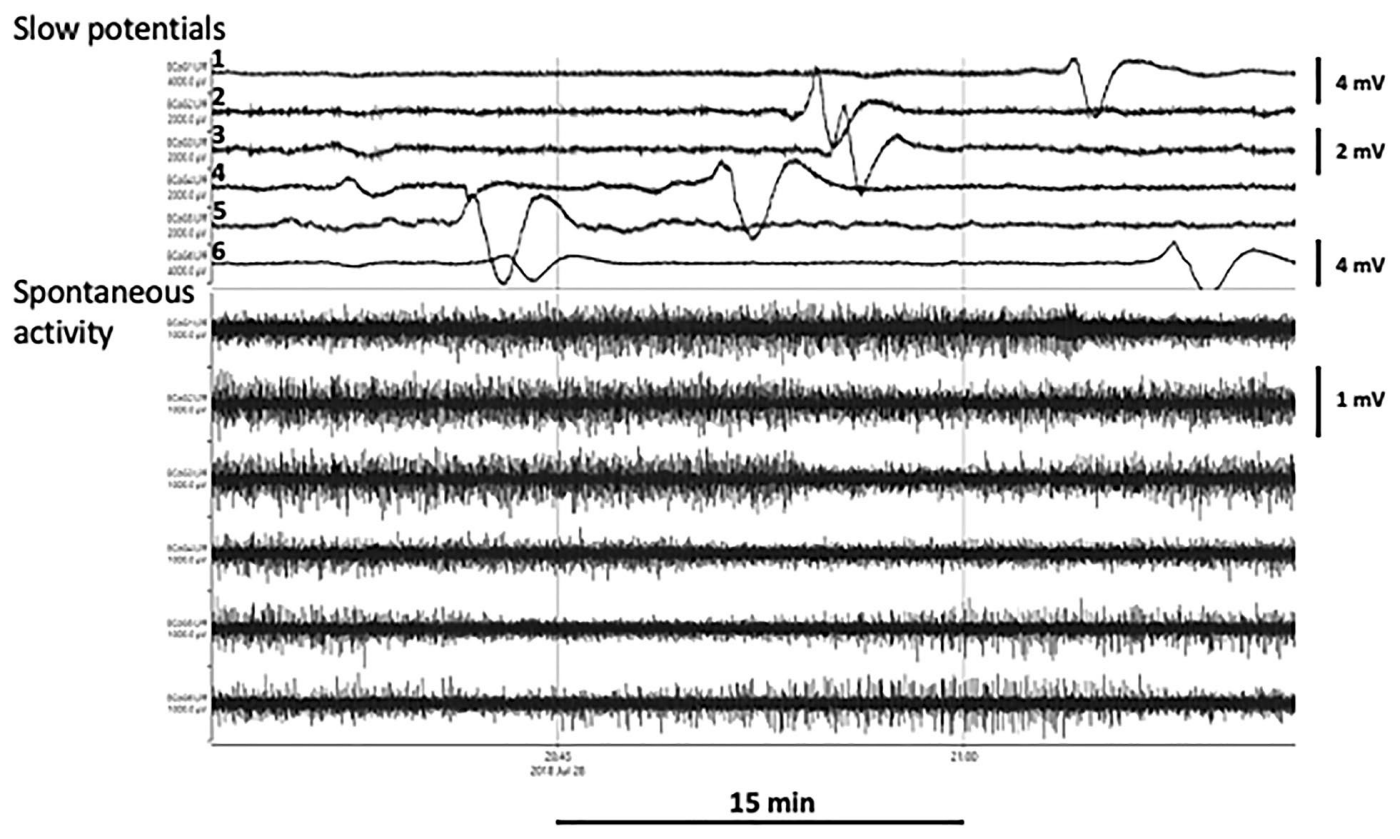
Example of a single spreading depolarization (SD), illustrating its passage through the underlying cerebral cortex from contact 5 to 1 by an irregular path (5, 6, 4, 2, 3, 1). Typical SPCs are seen when the raw DC-coupled recording is filtered at 0.005–0.5 Hz and depressions of spontaneous activity are observed in the conventional band of 0.5–45 Hz. Amplitude scales (to right side) for slow potentials are larger (4 mV) for channels 1 and 6 than the 2 mV for channels 2–5. The scale for spontaneous activity applies to all channels. A second SD appears on channel 6 toward the end of the period shown

Each channel of raw full-band (DC to Nyquist) data is split into low (0.005–0.5 Hz) and high (0.5–45 Hz) wavebands. Figure 1 illustrates the waveforms from the six channels in a series of SDs after filtering into two frequency bands, both as presented to the detector software and as visualized by the investigator listing SDs manually. The key low-level features are a reduction of the amplitude envelope (spontaneous activity) in the high-frequency waveband and a biphasic or triphasic transient of duration approximately 5 min, known as a slow potential change (SPC), in the low-frequency band.

The detector software seeks both types of low-level events, and SPCs are then linked first with any coincident suppression events in the same channel before being combined with nearby SPCs from other channels as the wave passes over the electrode array to create a high-level event. If none of the SPCs in an event are linked with a suppression, the event is classified as an artifact. If SPCs are linked with the onset of a suppression, the software will classify the event as a potential CSD, whereas SPCs that coincide with already suppressed high waveband data are given an ISD classification. A CSD/ISD classification is used when there are both new and existing suppressions in different channels. SPCs occurring simultaneously on several channels are classed as artifact.

The event combination and classification process uses a confidence system to handle uncertainty in the decision-making process. Each low-level feature has an associated confidence value, scored out of 100, representing the strength of the suppression or clarity of the SPC. These values then propagate through the event combination process, with high-level features assigned confidence values based on the number and confidence of the low-level features from which they are derived. Other low-level features, such as movement detected by accelerometer (not implemented in the patients reported here) or the presence of bad or missing ECoG channels, also affect the confidence value.

During manual assessment of ECoG data, stereotyping [2] is a key method in distinguishing between genuine SDs and similar-looking artifacts. Although the SPCs will vary significantly between patients and across channels within a patient, SPC shapes within a channel and the sequence/intervals of SPCs across channels will typically repeat in a stereotyped manner when multiple SDs from an individual patient are compared. The automated analysis uses a neural network-based pattern-matching algorithm [20] to compare the SPC shapes in each candidate event with every other candidate event and constructs a similarity matrix. A hierarchical clustering algorithm [21] uses this similarity matrix to split the potential events into groups that are mutually similar. Events in the major clusters are more likely to be genuine SDs, and so their confidence values are increased, whereas the confidence of the outliers, which are more likely artifacts, is decreased.

**Fig. 2:**
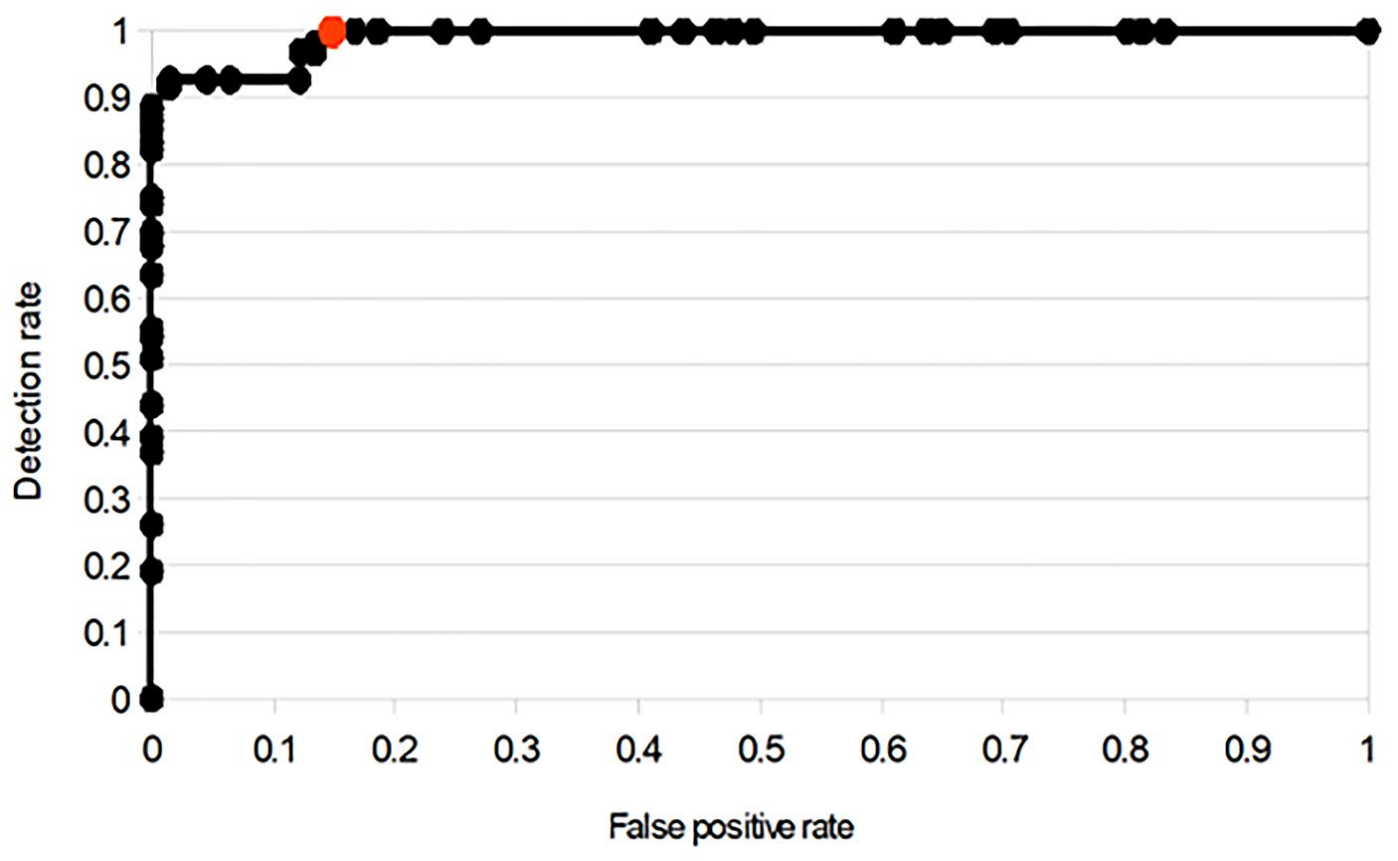
Receiver operating characteristic plot showing the change in detection rate and false-positive rate. Confidence threshold values from 0 to 100 are used to partition a list of potential events into artifacts and SDs. Because this plot only focuses on the ability of the confidence system to distinguish between genuine SDs and artifacts, perfect detection here does not correspond to perfect detection overall, because not all SDs are identified as potential events. Results for Fig. 2 are derived from 64.6 days of development data from 21 patients at 2 hospitals, with Neuromonitor classifications compared with ground truth files compiled manually by AJS. This data set was used to select a threshold level of 30, shown as the red data point, for use in the Neuromonitor software, which gave maximum sensitivity while limiting the number of false positives. There is no overlap between this earlier development data set and the present validation data set reported here

Lastly, a threshold is applied to the final confidence values to partition the potential events into SDs and artifacts, with the choice of threshold providing control over the sensitivity of the overall detection process. Figure 2 shows that the receiver operating characteristic curve, calculated by using the development data set, as the threshold is increased from 0 to 100. A threshold of 30, illustrated by the red dot, resulted in the best balance between sensitivity and specificity and was used by the Neuromonitor software for the validation testing reported in this article.

### Depiction and Software Grading of Detected SD Events

**Fig. 3:**
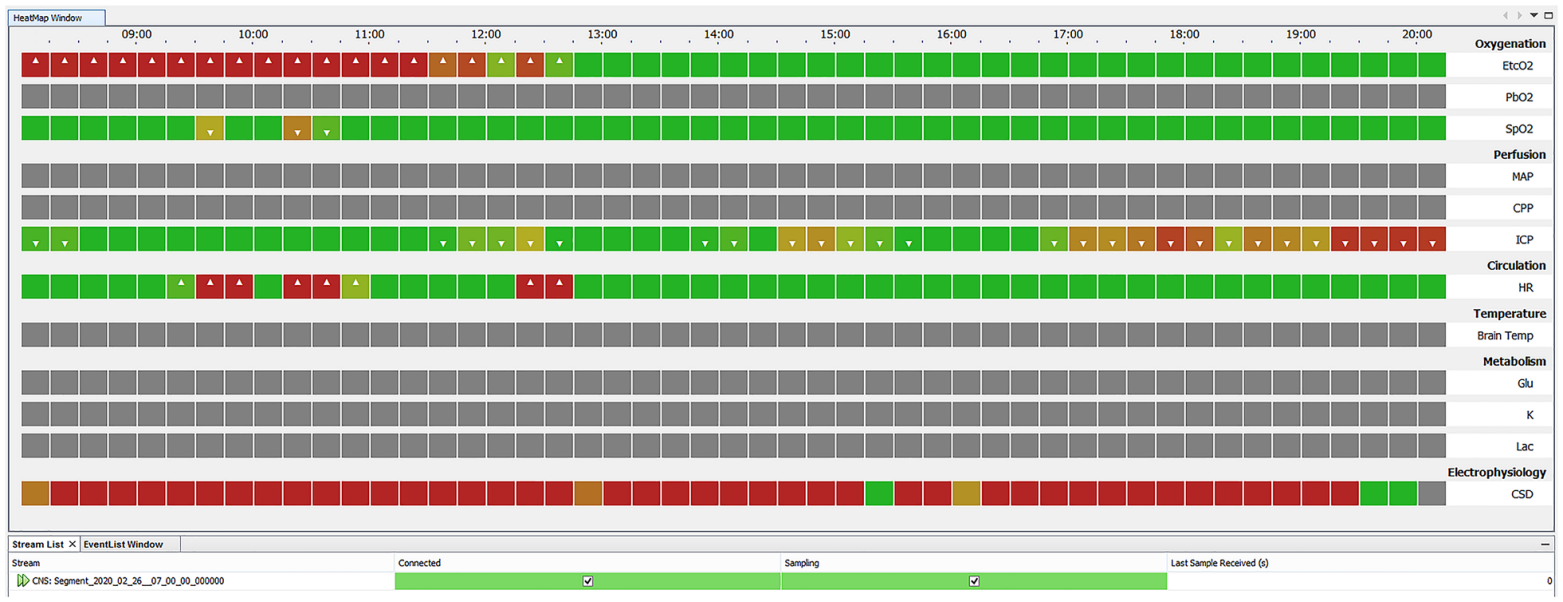
Example of the default Neuromonitor display, illustrating an example of 12 h’ processing. Each square block represents the grand average of 15 one-minute means of the variable. Where the grand average lies within the clinician-defined normal range, the block is rendered green or shaded up through orange to brown to red in proportion of severity of deviation. A white arrow in a square indicates whether the variable is above or below the specified range. There is an option for the user to vary the color scale in the case, for example, of a patient known to be hypertensive. Black rows indicate no signal is available from the source monitor. The three gray-colored lines represent a software placeholder for previous and future implementations of online sampling of metabolic variables in cerebral microdialysate. The essentially continuous succession of red squares on the lowermost line (electrophysiology: CSD) indicates a prolonged sequence of rapidly repeating SD events (a prolonged and intense cluster) of serious pathogenic potential [13]. Please see Fig. 4 for an example in which the detections are time marked to the ECoG events and to investigator ground truth. CPP, cerebral perfusion pressure; EtCO2, end-tidal CO2; Glu, brain tissue glucose; HR, heart rate; ICP, intracranial pressure; Lac, brain tissue lactate; MAP, mean arterial pressure; PbO2, brain tissue partial pressure of oxygen; SpO2, systemic oxygen saturation %

**Fig. 4:**
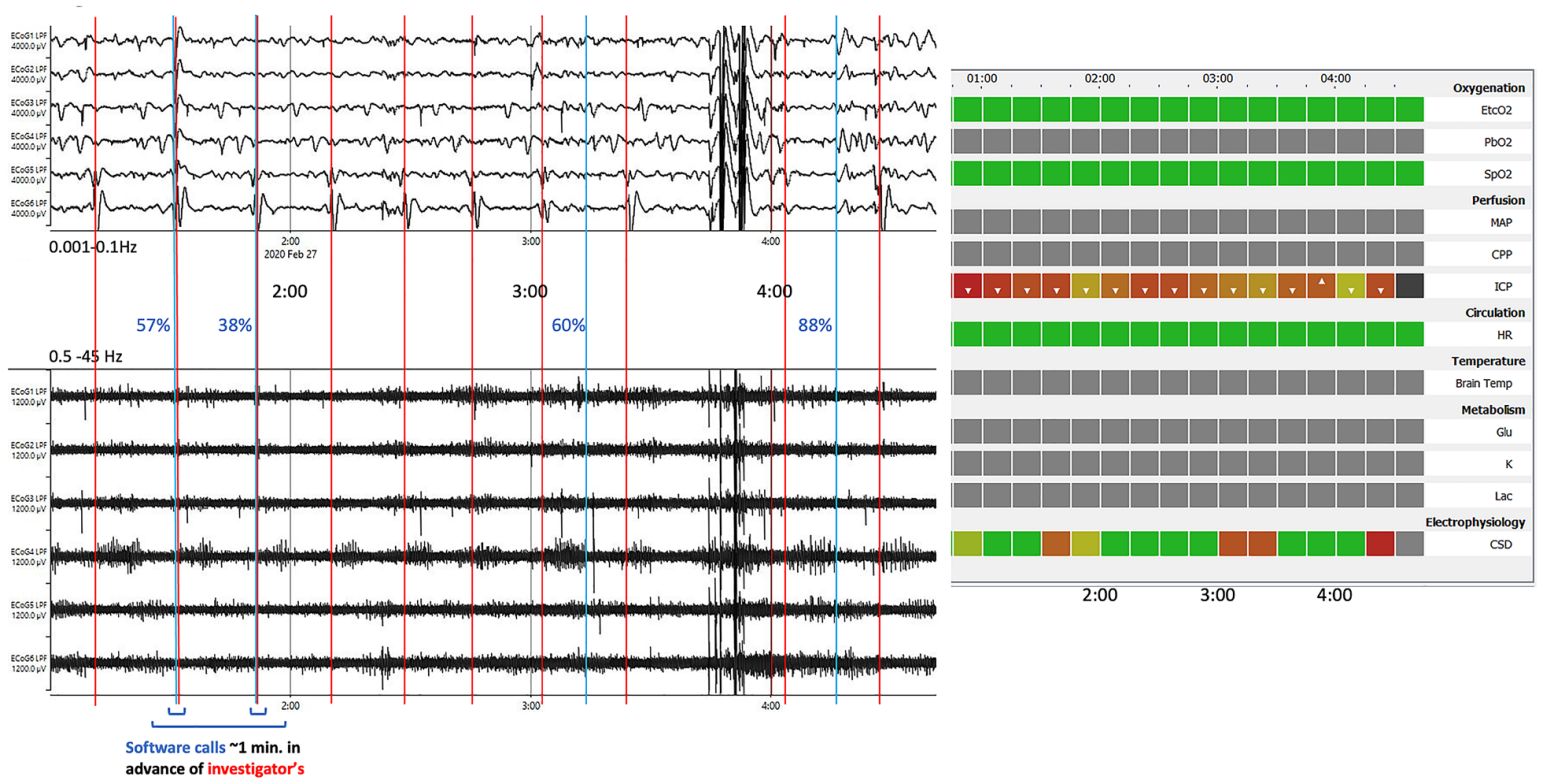
Example of ECoG data and corresponding software heat map output in one patient in which there was a poor match between software and investigator calls for SDs (Cincinnati No. 5; see also Fig. 5, point C, and Discussion). Top six ECoG traces are filtered for low frequencies (0.005–0.5 Hz) and lower traces for high (0.5–45 Hz) wavebands. Timings of investigator’s SD calls (red) and software calls (blue) are shown, with software percentage confidence values adjacent. At 1:31 and 1:51 a.m. there was close agreement on the calls, although software confidence was only moderate or low. Note that at 3:13 a.m., the single software SD listing is represented on the heatmap by two squares (3–3:30 a.m.) because the duration of the event straddled both 15-min sample blocks (see also video in the Supplementary Material). CPP, cerebral perfusion pressure; CSD, cortical spreading depolarization; EtcO2, end-tidal CO2; Glu, brain tissue glucose; HR, heart rate; ICP, intracranial pressure; Lac, brain tissue lactate; MAP, mean arterial pressure; PbO2, brain tissue partial pressure of oxygen; SpO2, systemic oxygen saturation %

A 15-min epoch for each variable is rendered green when no event is detected and red when one or more SD events are detected, with high confidence within the 15-min sample period (see lowermost trace in Figs. 3 and 4). Intermediate colors are shaded in proportion to confidence in the detection and may be increased toward red in retrospect (or revert to green) as confidence grows by stereotyping or diminishes subsequently as described above. At any time within the time range of the heat map, the user can select a given epoch on the CSD line and so obtain a graphic of the ECoG time series data, both SPCs and spontaneous activity, on which a particular detection is based. When listing an event, the software also logs the confidence in the detection, as described above, and characterizes the events as CSD, ISD (widely believed to carry greater pathogenic potential than CSD [12]), or CSD/ISD. Criteria for these designations are listed in the Appendix.

### Assessment of Detector Performance

In addition to its principal planned application (the capacity to detect and record SD events in real time at the bedside), it should be noted that precisely the same Neuromonitor executable is also capable of processing archive data (at a throughput rate of 24 times real time). The design of the present validation study is retrospective, in that some data were acquired and the ground truth scoring of data, as the reference standard, was conducted before the definitive software version was finalized. All data were subsequently processed by the final version to assess its performance relative to the manual scoring. Typically, a record or archive of some 24 h, but on occasion less than 24 h, was submitted as one single data set—a CNS310 archive file—to investigator review and later to Neuromonitor. After screening (please see Results), a total of 91 files were available from 18 patients (KCH = 13, UC = 5). Two experienced investigators manually scored the SD events, each from their own center’s data, and generated a ground truth data set from review of each file using CNS Reader (Moberg Solutions, Inc.), applying the criteria of Dreier et al. [10].

Each event reported and characterized by the software in a given record was checked against the corresponding time on the ECoG multichannel time series (in both frequency bands) and was designated a true or false positive in a comments column in the software output text file. When the investigator found an event with no corresponding software-detected event, this was entered in the file as a false negative in its own extra row. Software-detected events were then entered into a spreadsheet, one record per row, with separate columns listing totals in that record, which were categorized by the software confidence levels: high, medium, or low (for CSD); ISD; or CSD/ISD. These were totaled in an additional column. An additional column listed the total of medium confidence events with a confidence of 60% (a value chosen empirically as a possible software confidence binary cutoff) or greater but less than high confidence (range 70–100%). Additional columns listed (1) the investigator’s count of all events (without subclassification for KCH data), (2) false positives, and (3) false negatives. The total of SD events (all levels of confidence, 30% and above, and unclassified by CSD/ISD) detected in each record was then compared with the investigator event count by using linear regression (unconstrained). Further analysis is described in the Results.

In a second analysis, we calculated sensitivity and specificity (expressed as false-positive rate on a scale of 0–1.0 for each of the 91 data points by using a 2 × 2 contingency table; individual data not submitted). True positives were defined as investigator-confirmed software SD reports aggregated for the sampling period (typically around 24 h). False positives were software-reported events rejected by the investigator over the same sampling period. False negatives were logged by the investigator adding a line to the software record timing the missed event and again aggregated over the same sampling period. We generated a value for true negatives as follows: because the maximum number of recurrent SDs per hour is approximately three, we posited that a true negative event would occupy 20 min of sampling time, and we calculated the number of 20-min periods not occupied by a verified SD as the number of true negatives for the record in question. Sensitivity was calculated for each record as the number of confirmed software detections divided by the sum of the confirmed detections (positives) and investigator detections missed by the software (false negatives). The false-positive rate was calculated over the duration of the record as the number of false positives divided by the number of 20-min periods not occupied by a verified SD, in other words “silent.” For calculation of sensitivity, this was not possible when the denominator (verified detections + software-missed detections) was zero. Thus, the number of calculations of sensitivity that was possible fell well short of those for the false-positive rate (always possible).

## Results

ECoG records of five anonymized patients from UC were scored by JAH and later processed through the SD detector in Neuromonitor. From KCH, anonymized records of 13 patients remained available to AJS after screening. Data sets were rejected or were not collected (1) when patients’ conditions deteriorated soon after return to the ICU and monitoring was judged no longer appropriate (*n* = 3), (2) when an artifactual ECoG event referable to nursing care was the sole feature in the trace (*n* = 1, discussed below), or (3) when SDs were restricted to a single ECoG channel (*n* = 2; please see Discussion). Typically, data were acquired continuously for some 24 h, depending on clinical requirements, such as interruptions for interval computed tomography scanning or other procedures. The number of records from a given patient would thus depend principally on the number of days of monitoring, and 91 data sets were available from 18 patients (KCH 13, UC 5; Table 2).

### Consistency of Investigators’ Ground Truth

In a brief check on interinvestigator consistency, event counts in the UC data sets by AJS were found to match very closely with those by JAH. We therefore relied on manual event counts by a single individual, especially in consideration of previous findings by Hartings et al. [22] of close interobserver agreement on manual scoring.

### Tabulation and Comparison of Software SD Counts Against Investigators’ Ground Truth

**Fig. 5:**
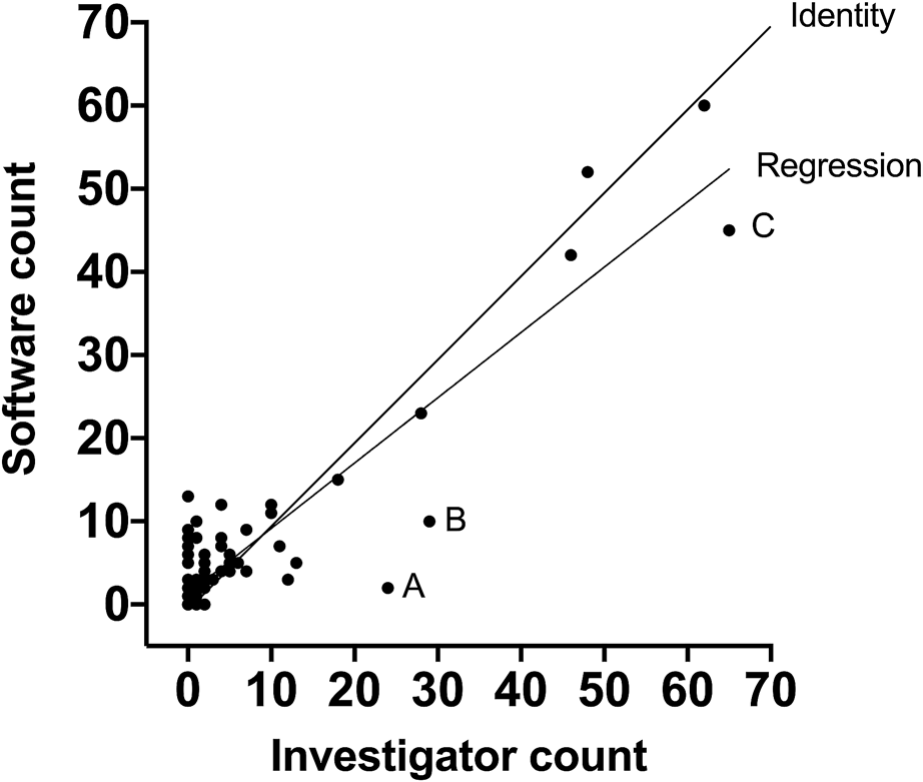
Scatterplot comparing the software (Neuromonitor) count of depolarizations (SDs, all types: CSD, ISD, CSD/ISD; dependent variable, ordinate axis) against the investigator manual count (independent variable, abscissa) by linear regression. The slope of the regression was 0.7855 (95% confidence interval: 0.7149–0.8561), significantly less than identity (labeled line). *Note*: (1) 27 records in which neither the software nor investigators saw any candidate event are superimposed at coordinates 0.0, and (2) there were a substantial group of false positives, again with many superimposed points, in which the investigator count was ten or less. Points labeled A, B, and C are outliers, reflecting significant underdetection by the software, and are considered in the Discussion

Among the 18 patients in the verification data set (Table 2), total duration of monitoring ranged between 29 h and 16 days. The total time sampled and analyzed was 1915.4 h. Each record was treated as an independent data set for analysis by Neuromonitor and by the investigators, yielding 91 data points for unconstrained linear regression analysis, with total event count (aggregate of all three patterns of SD events—CSD, ISD, and undetermined whether CSD or ISD—and regardless of the confidence percentage in the call reported by Neuromonitor) as the dependent variable and investigator’s ground truth as the independent variable. The slope of the regression (Fig. 5) was 0.7855 (95% confidence interval [CI] 0.7149–0.8561); thus, a slope of 1.0, the line of identity (shown), lies outside and above the 95% confidence limits, and the regression data point to an overall sensitivity of 79%. *R*^2^ was 0.8415. There was a significant positive intercept on the *y* axis (1.30; 95% CI 0.38–2.22).

We noted visually, first, a striking cluster of points above the regression and equality lines when the investigator’s count lay below *n* = 11, suggesting a cluster of false positives and a clear trend toward lower sensitivity above this count. We also noted the finding of 27 records among the 91 (29.7%) in which neither the SD detector nor the investigator recorded any SD of any pattern during an aggregate sampling time of 431.2 h (22.5% of total sampling time); clearly, all of these points plotted to coordinates 0,0 on Fig. 5.

### Sensitivity and Specificity Analyses

Sensitivity was expressed on a range of 0.0–1.0, and the required nonzero value for the denominator of the expression TP/(TP + FN)^2^ was available in 48 of 91 records. In 27 such records, sensitivity was at the maximum of 1.0, indicating that all investigator-detected SDs were also captured by the software. The median value was 1.0, the lower 95% confidence limit was 0.83, and the lower 25th percentile was 0.66. However, in 14 of these records, it was noted that the sensitivity of 1.0 derived from a single correctly detected SD.

The false-positive rate (specificity) was calculated as described above and also expressed on a scale of 0.0–1.0. Values were available from all 91 records. The median value was 0.0090, with an upper 75th percentile of 0.039 and an upper 95% confidence limit of 0.014. The maximum individual value was 0.19.

### Comparison of Software Confidence Level 60% vs. 30%

In light of the initial experience of a high number of false positives with confidence values between 30 and 60%, we proposed that 60% confidence might be a more clinically useful threshold to apply as a definition of an SD-candidate event. We therefore examined the effect of increasing the original 30% confidence threshold to 60% on the number of events reported in each software record that exceeded the investigator’s count. At 30% confidence, there were 40 records in which the software count exceeded the investigator’s; notably, only in one such record did the investigator count exceed 10 (Fig. 5). At 60% confidence, the figure of 40 fell to 16. We then reexamined the overall regression equation, restricting software SD calls to those with confidence of 60% or greater and found a slope of 0.5520 (95% CI 0.4979–0.6060), indicating considerable loss of sensitivity; the intercept of the line of regression on the *y* axis (0.36; 95% CI − 0.4534 to 0.9779) was no longer significantly above zero.

To test whether the excess of software SD counts at low investigator total counts reflected a greater proportion of low-confidence (less than 60%) SD calls than greater than 60% confidence in the calls exceeding those by investigators, we expressed the number of individual false positives with confidence percentages between 30 and 60 as a percentage of all software calls for the record in question. There were 37 such records in which the total software SD count exceeded investigators’: the median percentage of low-confidence calls was 63%. There were 27 records in which the raw software count was equal to or less than investigators’; here, the percentage of low-confidence calls was 11%, significantly lower (Mann–Whitney 2-tailed *U*-test = 257; *p* = 0.0004). Thus, it appears that the number of software counts in excess of investigators’ when SDs are infrequent is due to a higher proportion of lower-confidence software calls in these circumstances. When we used the 60% confidence threshold, the number of records in which software count exceeded investigators’ fell from 40 to 16. (The remaining 27 records with no events recorded either by software or by investigators were not relevant to this exercise.)

Considering now the less populated area in Fig. 5 in which software and investigator counts were in the higher ranges (investigator count greater than 10, *n* = 11), we examined the overall results on the basis of all software SD detections, i.e., with a confidence of 30% and above. In all but one of these records, the software count was lower than that of the investigator, especially in the three points labeled A, B, and C in Fig. 5. Possible explanations for these outliers are listed in the Discussion.

### Experimental Evaluation of Fraction of Inspired Oxygen Bolus as a Source of False Positives

**Fig. 6:**
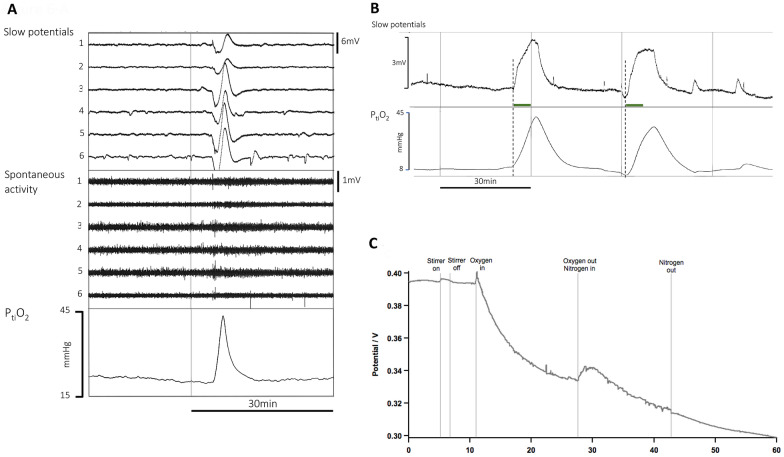
Demonstration of an electrochemical cause for simultaneous multichannel slow potential changes caused by a bolus of 100% inspired oxygen. **a** Example of 100% fraction of inspired oxygen (F_i_O_2_) bolus and concurrent hyperoxic ECoG transient seen in the excluded patient. A high-voltage (true DC values attaining 40 mV) slow potential transient is seen across all channels in the top bank of signals. The middle bank of signals (filtered to select 0.5–45-Hz activity) shows the frequency range within which depression of spontaneous activity would typically be seen with a SD. No such depression of activity is noted here. The bottom trace shows increase in brain tissue oxygen tension (P_ti_O_2_) coupled to the F_i_O_2_ bolus. Future developments in the detector algorithm will seek to identify such hyperoxic ECoG transients specifically and separate from SDs. **b** The effect of F_i_O_2_ bolus on slow potential ECoG and brain tissue oxygen. Upper: The DC output (positive potentials displayed upwards) of contact 4 on the ECoG strip of patient London-11. Similar signals were seen simultaneously on all electrodes of the strip. No change in spontaneous activity (0.5–45 Hz) was seen (data not shown). Lower: P_ti_O_2_ signal recorded at the same time using the Raumedic Neurovent–PTO combined probe for ICP and P_ti_O_2_. The green bars indicate F_i_O_2_ bolus delivered prior to airway suction or other nursing maneuvers. **c** The effect of oxygen bubbling on the open-circuit potential of a platinum electrode. Potentials measured vs. Ag/AgCl by the high-impedance CHI system. System voltage was stable for 1 h before the experiment. Oxygen and nitrogen bubbling were each for 15 min. The true DC voltage change seen for the CHI system is a downward shift of 62.0 mV from a stable open-circuit potential of 0.396 V (vs. Ag/AgCl). The shift is not caused by mass transport effects, and activating a magnetic stirrer had no effect. For the voltmeter system, similar effects were seen (data not shown). The open-circuit potential was lower at 0.147 V (most likely because of the higher input bias currents of the voltmeter being satisfied by oxygen reduction), and the downward voltage shift was 23.0 mV. Replacing the oxygen bubbling with nitrogen bubbling of the same intensity caused a transient reversal in open-circuit potential (+ 17 mV CHI, + 77 mV voltmeter). Very similar effects were also seen when phosphate-buffered saline was used (data not shown)

In some patients, a P_ti_O_2_ probe was placed near the ECoG strip at craniotomy for clinical indications. A noteworthy incidental finding in this study that led to the exclusion of one patient was the occurrence of a number of low-frequency transient events occurring simultaneously on the majority of electrodes on a strip and immediately following a fraction of inspired oxygen (F_i_O_2_) bolus delivered prior to airway suction or other nursing maneuvers (Fig. 6a, b). There was no associated change in the amplitude of the spontaneous activity (0.5–45 Hz) signal, but these events were reported by Neuromonitor as SDs. We posited that such a low-frequency transient, simultaneous at multiple contacts, could have an electrochemical explanation; if the platinum ECoG disk was poised at a negative potential compared to the reference (as is possible), it could be capable of conducting oxygen reduction. A change in oxygen concentration would reduce the charge transfer resistance, allowing the 2-mV voltage to drive nanoamp current into the amplifier. This is possible because the high-input impedance of the electrophysiological amplifiers is 100 MΩ. Such effects can be seen with pH electrodes if a voltmeter, rather than an electrometer-grade amplifier, is used.

To test this proposal we conducted a laboratory experiment. At room temperature (25˚C), artificial cerebrospinal fluid solution (aCSF) containing KCl (2.7 mM), NaCl (147 mM), CaCl_2_ (1.2 mM), and MgCl_2_ (0.85 mM) was placed in an open beaker. Two commercial platinum (Pt) electrodes (1.2 mm diameter; CH Instruments, Austin, TX) and two reference electrodes (Ag/AgCl/3M KCl; BASi, West Lafayette, IN) were placed in the aCSF. One Pt electrode/reference electrode pair was connected to an electrometer-grade high-impedance amplifier input (CHI-650a in open-circuit potential mode, input impedance greater than 10^12^ Ω; CH Instruments). The second pair was connected to a lower-input impedance voltmeter (Amprobe 5XP-A digital multimeter, Amprobe, Everett, WA, USA) input impedance greater than 1 MΩ). This was chosen because it was noticed that the electrophysiology inputs for the electrophysiology amplifiers, although classed as high impedance, were in fact less relative to the electrometer, at 100 MΩ. This of course is separate from their isolation from earth required for patient safety. After a 2-h period allowed for stabilization, oxygen was bubbled into the solution for a period of 15 min, followed by nitrogen for 15 min, followed by another period of stabilization. The results are shown in Fig. 6c. The results are qualitatively consistent with what is seen in Fig. 6a when we take into account that the slow potential ECoG data are presented in the standard electrophysiological convention of hyperpolarization being an upward voltage shift. Hence the 3-mV slow potential ECoG shift in Fig. 6b (DC coupled in Fig. 6b rather than AC, as in Fig. 6a) represents in absolute terms a reduction in voltage. The P_ti_O_2_ value in Fig. 6b shifts by 37 mm Hg. We estimate that in vitro, air-saturated aCSF has approximately 118 µM dissolved oxygen. This increases approximately fivefold when bubbled with pure oxygen. Thus, it would appear that the basis of the ECoG SPC associated with an F_i_O_2_ bolus is electrochemical. Our initial experience is that this artifact can be recognized in AC-coupled ECoG traces (filtered to select SPCs) as rapid-onset large amplitude transients highly synchronous in most or all channels (Fig. 6a, b). We shall seek to edit the software SD-detection algorithm to reduce further the risk of false positives from this source of artifact.

## Discussion

To our knowledge, this is the first report of automated detection of SDs occurring in patients with acute brain injury; we intend that it should serve as a basis for further development. Such a development is timely because of increasing recognition by neurointensivists of SDs as a priority for clinical research [23], and in many centers, SD monitoring has been adopted as a standard of care. In the case of TBI, some 60% of patients undergoing emergency craniotomy with placement of a subdural ECoG strip prior to closure experience SDs, and in such patients, the frequency of SDs can be high [13]. Any sequence of three SDs occurring within a period of 2 h is often defined as a cluster, and there is clear evidence that such clusters of CSDs (please see footnote in Introduction), characterized on first occurrence by hyperemic neurovascular coupling and relatively quick recovery to baseline ECoG activity, tend to develop into sequences of ISDs. These ISDs, by contrast, are characterized by persistent ECoG depression, more vasoconstrictive neurovascular coupling [24–26], and prolonged duration of SPCs, indicating progressive failure of tissue to repolarize [13]. Here, 6 of our 18 patients experienced at least 24 SDs over the course of 24 h, and in one, 113 SDs were recorded over 2 consecutive 24-h periods; in this patient, a trend toward ISD in the second 24-h period was detected by the software, although incompletely reported (point C on Fig. 5). The same pattern of deterioration from CSDs to ISDs and, ultimately, to terminal depolarization has been described in patients with aSAH [27–30].

This software effectively addresses the need of neurointensivists for a simple method to reveal the presence of significant SD activity. Although the 95% CI for the slope of the regression of the Neuromonitor event counts versus the investigators’ ground truth lies below unity, indicating mild insensitivity of the detector, Fig. 5 demonstrates that when SDs are frequent, they will nevertheless be detected—if not in total count, then certainly still in reliable qualitative terms. Conversely, review of the regression plot (Fig. 5) demonstrates that there were no instances of high numbers of false-positive reports such that would risk treatment errors: the highest false-positive rate in the entire study was 0.19 (range 0–1.0), and among data in which the investigator count was higher than 10, there was only one instance in which the software count exceeded the investigators’: by 8% (52 vs 48).

What conclusions can be drawn from the parameters of the regression plot (Fig. 5) (software SD count = [0.786 × investigator count] + 1.298)? The large number of false positives in which the investigator count lies below ten seems likely to account for the positive bias of the regression intercept. There was a significantly higher proportion of lower-confidence software calls in this group of 37 comparisons than in the 27 records in which the raw software count was equal to or less than the investigator count; here, the percentage of low-confidence calls was 11%, significantly lower (Mann–Whitney 2-tailed *U*-test = 257; *p* = 0.0004). When the low-confidence software calls were discounted, the intercept was no longer significant; we inferred from this that the large number of false positives (in which the investigator count was ten or less) might have been responsible for the positive bias (+ 1.298) toward the software count in the regression. These false positives represent occasional events, often scattered over a period of some 24 h; the maximum in any one patient was 13 events, well dispersed. We attribute these overdetection errors to lack of a stereotype, deriving from the absence of serial repetitions of similar SDs. In this context, we reemphasize that in nearly 30% of the records (22% of sampling time), no SDs were seen by either the investigators or the software.

Three outlying points (Fig. 5, points A, B, and C) appear to be particular sources of low sensitivity, and we have examined these data sets in some detail. One theme emerges as a trend for ECoG signal amplitude to deteriorate over the course of days, more noticeably in the 0.5–45-Hz bandpass filtered (spontaneous activity) domain. The software makes use of stereotyping as an aid to identification of an SD, thus learning from early events (as is sometimes evident in a rise after 15–60 min in reported probability of an event being an SD). Progressive loss of recovery of the higher-frequency band amplitude after a CSD, reflecting progression toward ISDs, entails departure from the stereotype, raising a risk of underdetection. In addition, in the data for point A (Fig. 5), we saw sudden and unpredictable sharp fluctuations in signal amplitude between a higher and a lower value, which we interpreted as movements of the electrode strip alternately into and then out of contact with the cerebral cortex. The underdetection of events reflected in point C was reviewed in particular depth. In the early hours of this record (Fig. 3), events occurred regularly (as widely observed [31, 32]) at 21-min intervals for 7 h, with precise matching of software SD detections and ground truth. Later, counting became more difficult because of overlap in time, with consecutive SDs present simultaneously at opposite ends of the strip, leading to underdetection. A second challenge in this data set was a period of heavy ECoG artifact lasting some 40 min; following this (Fig. 4), although a regular SD pattern returned at a frequency similar to earlier, the amplitude and morphology of events had changed, and remodeling of the previous stereotyping was not sufficiently rapid in this situation.

### Utility of SD Detector Software in Clinical Practice

Thus, the parameters of the regression appear to reflect a high incidence of false positives when SDs are sporadic and perhaps of varying ECoG morphology and a risk of undercounting at high SD frequencies. How should the findings and their interpretation guide use of the system in clinical practice, when a clinical team will rely largely on the current heat map display rather than on a printout text summary of a completed period of several hours? The significance of a first flag of an SD will depend heavily on the confidence of the software (denoted by its allocated color: range green to red), but it seems unlikely on its own, even with a high-confidence tag, to prompt any immediate response at the bedside. Because the display is updated at 15-min intervals, it is the subsequent chain of events that will determine any clinical response. The occurrence of three or more SDs within a period of 3 h or less has been defined as a cluster [33] and is believed to carry an increased risk of lesion progression. The heat map display as currently configured will display such clusters. However, at higher SD recurrence rates of one to three per hour, and as explained above and for differing reasons, the heat map display may either under- or overrepresent SD frequency. Our results suggest that the text record of occurrence will not overrepresent higher occurrence rates of genuine SDs, but it is not currently available for display on the heat map.

A user strategy of regularly discounting SD calls of low confidence by the software as it is currently configured carries a clear risk of undercounting what may be true events. Future work might justify revisiting this issue.

In an effort to improve further the performance of the software, we identify a number of opportunities to optimize our software and clinical routines. First, the detection algorithm was developed on data sets derived from a sequential bipolar montage of the subdural strip, and it hence relied in part on the (inherent) phase inversion of the SPC profile in adjacent channels. For the future, we shall apply a different version of the detector that was developed for use on unipolar montages. Second, and subject to agreement among our surgical teams, we shall introduce and assess, as soon as possible, a combination of changes in surgical and recording procedures (manuscript in draft) to ensure more stable contact of the electrode strip with the cortical surface. Third, if improvements in signal to noise ratio are maintained, we expect to increase the sensitivity of the detector to SD events restricted to one or two channels (such as those that were not always detected by the current prototype version of Neuromonitor). Fourth, use of an accelerometer will reduce false positives, whereas the P_ti_O_2_ signal can increase sensitivity and is especially useful for detection of ISDs. Finally, work will be required to reduce the risk of false positives from F_i_O_2_ boluses. The impact of these proposed changes will require reassessment in comparison with the results reported here.

In this work, we seek to provide, principally, a means of detecting and counting SDs and depicting their frequency in an easily understood format, but the display routine adopted also offers a simple visual means of detecting changes in critical systemic variables that might have precipitated SDs. The current sensitivity of the system of 80% is certainly sufficient to detect and highlight clusters of SDs that are believed to carry particular pathogenic potential by virtue of their frequency [12, 13]. This offers the possibility of distinguishing different sequences of deterioration and interrupting them. Because the system provides continuous monitoring for SDs and immediate detection, it also obviates the need for either the continuous presence at the bedside of a clinical neurophysiologist or the need for periodic retrospective specialist reviews, with a consequently delayed therapeutic response. A very positive, qualitative finding is that the availability for the first time of a simple display depicting frequency of occurrence of SDs has engendered increasing interest from the bedside clinical teams and prompt discussion among them of the need for a therapeutic response to, especially, a cluster of SDs. Similarly, the facility to visualize on the heat map screen a concise representation of adverse changes in systemic variables in their own right, as well as their relationship to SD occurrence, is beginning to prove valuable, as it prompts and informs discussion of causality and therapeutic options. As a corollary, we anticipate making use of the Neuromonitor heat map display and its event-listing in text format to facilitate clinical research projects in which SD frequency is an end point; in this setting, in which more rigorous listing of SD events may be required, an investigator may, for example, opt to focus manual review of SD-candidate events listed by the software with confidence less than 70%. In the longer term, the algorithm that has been applied to detect the electrocorticographic waveform of SDs in postoperative patients is potentially capable of adaptation to novel waveforms deriving from, for example, any future technology targeted with adequate reliability at noninvasive detection of SDs from the scalp.

### Limitations

This report can be regarded as incomplete in that we identify above a number of possible improvements to our procedures, as well as to the source code itself, and the current data can be seen as a preliminary reference standard against which future developments can be assessed. In particular, there is a need to understand and improve the reduced sensitivity that we found. With further study, we shall aim to resolve whether this is systematic or merely reflects underdetection in certain circumstances that can be recognized and taken into account. Comparison of the case mixes in the development (Table 1) and validation (Table 2) data sets indicates a significant number of patients in the development set undergoing decompressive craniectomies for MHS, compared to only one in the validation set. Because the number of investigator SD calls in these (development set) patients with MHS was low (thought to be due to location of the strip on core tissue), the information gained from this group was low; however, aside from these cases, the sizes and compositions of the two groups are similar. We were unable to confirm that there is a precise match between the input frequency bandwidths to the detector software used in the development set and those used in the validation set. Nevertheless, this report describes the performance of the current software.

In view of the pathogenic potential of ISDs, it is unfortunate that the capacity of the software to identify specifically these events was not more fully tested (Fig. 5, point C). Although some ISDs were detected, they were significantly underreported. The second data set, in which multiple ISDs were seen, is illustrated in the Supplementary Material and is more positive; here, the detection rate for 62 true events (unspecified for CSD, ISD, or CSD/ISD) was 90%, and 63% for fully matched detections, with three false positives during a total sampling time of 31 h. We conclude from these limited data that, as the software currently performs, some caution will be required in clinical use when an unexplained fall in SD frequency is seen on the heat map.

A further issue not addressed at this stage of development is the reliance of the heat map depiction of SDs on a pseudo-rainbow color code to indicate software confidence in the SD call; this is likely to be of little use to a color-blind individual, but the issue is being addressed.

Possibly related to reduced sensitivity and specificity here, but applicable to any undertaking to monitor for SDs, scrupulous attention to careful and effective placement of ground and reference contacts and their maintenance is essential.

## Conclusions

We show with this evaluation of a prototype software that it is possible to detect the presence of SDs in clinical ECoG recordings in real time by using an automated procedure. The output of this automated scoring is close to that of experienced assessors and sufficiently accurate for clinical utility. We have identified means of addressing slight undersensitivity in the current implementation of the detection method.

### Supplementary Information

Below is the link to the electronic supplementary material.

Supplementary file 1. Cincinnati No. 5: Figure 4 details of investigator and software calls during a period of repetitive ISDs late in the record. The low software detection rate in this example (point C in Fig. 5) is accounted for by underdetection after a period of disturbance at around 10:30 p.m. and is addressed in the Discussion. The video (time lapse) illustrates the entire Neuromonitor heat map capture (from the archive of) a 24-hour period of monitoring. By choice, not all available channels from the archive were sampled. By pausing the replay, it is possible to verify that a 15-minute period initially assigned clear of SDs or containing low-confidence SDs may be recategorized definitively soon afterward.(MP4 15942 kb)

Supplemental file 2. Software detection of ISDs and CSD/ISDs. Example of output from the (prototype) validation tool within the Neuromonitor software, applied here to a rare data set containing 61 ISDs or CSD/ISDs among 62 SDs in a record of 31 hours’ duration. The poorer performance (as reflected in mismatches) later in the record is currently attributed to undercounting when SD events recur in close succession. (HTML 8 kb)

## Appendix

CSD: If an SPC can be matched with an HPF suppression.

ISD: If an SPC can be matched with a channel that is already suppressed.

Artifact: If no SPCs can be matched with either a suppression or an already suppressed channel.

CSD/ISD: If some SPCs are matched with an HPF suppression and others are matched with an already suppressed channel.

## Footnotes

1 In this text and where necessary, we classify SDs as either cortical spreading depolarization (CSD, as is accompanied by transient, 5-15 minute suppression of electrocorticography (ECoG) amplitude, and hyperemia) or isoelectric spreading depolarization (ISD), in which the predepolarization ECoG amplitude is already abnormally low or fails to recover after the ISD. ISDs are frequently accompanied by a vasoconstrictor response in the cortical microcirculation. The term SD denotes either.

2 TP: True positive, confirmed; FN: False negative, seen only by investigator.
