# Supplementary material for: Development and Evaluation of a Method for Automated Detection of Spreading Depolarizations in the Injured Human Brain
Source: Neurocrit Care. 2021 Jul 26;35(Suppl 2):160–75. doi: 10.1007/s12028-021-01228-x (PMC8536628; doi:10.1007/s12028-021-01228-x)
Supplement: Supplementary file 2 — Supplemental file 2. Software detection of ISDs and CSD/ISDs. Example of output from the (prototype) validation tool within the Neuromonitor software, applied here to a rare data set containing 61 ISDs or CSD/ISDs among 62 SDs in a record of 31 hours’ duration. The poorer performance (as reflected in mismatches) later in the record is currently attributed to undercounting when SD events recur in close succession. (HTML 8 kb) [file 12028_2021_1228_MOESM2_ESM.html]

Search Results


Results written at 2021-02-01 14:00:10

Summary of detection results
Date: 2019-01-15

| Event Found | True Event | Match Type | Time | Conf |
| ISD | CSD | Mismatched | 13:37:12 - 13:52:22 | 56 |
| ISD | ISD | Matched | 14:24:26 - 14:42:59 | 52.9096 |
| CSD/ISD | CSD/ISD | Matched | 14:52:28 - 15:08:55 | 76.5854 |
| ISD | ISD | Matched | 15:23:43 - 15:35:32 | 49.9903 |
| ISD | ISD | Matched | 15:55:44 - 15:59:30 | 66.5 |
| ISD | ISD | Matched | 16:19:48 - 16:24:39 | 66.2019 |
| CSD/ISD | ISD | Matched | 16:46:06 - 16:53:48 | 83.843 |
| CSD/ISD | CSD/ISD | Matched | 17:07:24 - 17:15:04 | 55.6619 |
| CSD/ISD | CSD/ISD | Matched | 17:25:23 - 17:35:10 | 68.25 |
| CSD/ISD | CSD/ISD | Matched | 17:53:24 - 18:05:26 | 76 |
| No Event | ISD | Missed | 18:26:59 - 18:41:59 | 0 |
| CSD/ISD | CSD/ISD | Matched | 19:00:48 - 19:12:06 | 64.9226 |
| ISD | ISD | Matched | 19:23:46 - 19:37:12 | 86.2972 |
| CSD/ISD | ISD | Matched | 19:59:01 - 20:12:56 | 85.1329 |
| CSD | CSD/ISD | Mismatched | 20:29:33 - 20:47:09 | 70.4123 |
| ISD | ISD | Matched | 20:55:07 - 21:06:50 | 57.2957 |
| CSD/ISD | CSD/ISD | Matched | 21:21:38 - 21:35:25 | 76 |
| CSD/ISD | CSD/ISD | Matched | 21:47:21 - 22:05:02 | 58.0379 |
| ISD | ISD | Matched | 22:13:49 - 22:23:02 | 66.5 |
| ISD | No Event | False Positive | 22:34:33 - 22:45:41 | 53.8923 |
| CSD/ISD | ISD | Matched | 23:05:23 - 23:22:31 | 94 |
| CSD/ISD | CSD/ISD | Matched | 23:32:49 - 23:43:33 | 62.491 |
| CSD/ISD | ISD | Matched | 00:01:50 - 00:15:25 | 66.5 |
| CSD/ISD | CSD/ISD | Matched | 00:30:48 - 00:40:32 | 76 |
| CSD/ISD | CSD/ISD | Matched | 00:58:30 - 01:11:14 | 68.25 |
| ISD | CSD/ISD | Matched | 01:25:44 - 01:35:54 | 61.7488 |
| ISD | ISD | Matched | 01:53:21 - 02:05:26 | 76 |
| CSD | ISD | Mismatched | 02:20:35 - 02:25:41 | 46.5 |
| ISD | CSD/ISD | Matched | 02:46:41 - 02:54:30 | 66.5 |
| No Event | CSD/ISD | Missed | 03:13:52 - 03:19:52 | 0 |
| ISD | ISD | Matched | 03:30:51 - 03:41:28 | 84.2161 |
| CSD | CSD/ISD | Mismatched | 04:12:21 - 04:32:34 | 88 |
| CSD/ISD | CSD/ISD | Matched | 04:35:32 - 04:53:19 | 76 |
| ISD | ISD | Matched | 04:57:18 - 05:13:49 | 76 |
| CSD/ISD | CSD/ISD | Matched | 05:18:55 - 05:33:38 | 76 |
| ISD | CSD/ISD | Matched | 05:40:29 - 05:53:27 | 76 |
| ISD | ISD | Matched | 06:05:23 - 06:12:53 | 76 |
| CSD/ISD | CSD/ISD | Matched | 06:28:55 - 06:34:05 | 72.2644 |
| No Event | ISD | Missed | 06:43:14 - 06:58:23 | 0 |
| ISD | CSD/ISD | Matched | 07:10:04 - 07:20:10 | 76 |
| CSD/ISD | CSD/ISD | Matched | 07:29:42 - 07:48:39 | 93.3903 |
| CSD/ISD | No Event | False Positive | 08:05:29 - 08:16:58 | 62.9057 |
| CSD | CSD/ISD | Mismatched | 08:43:08 - 08:47:43 | 61.5 |
| CSD/ISD | CSD/ISD | Matched | 09:08:24 - 09:19:36 | 57.8588 |
| CSD | CSD/ISD | Mismatched | 09:42:46 - 09:47:31 | 54 |
| No Event | ISD | Missed | 10:11:50 - 10:26:50 | 0 |
| No Event | CSD/ISD | Missed | 10:30:58 - 10:45:58 | 0 |
| No Event | CSD/ISD | Missed | 10:58:54 - 11:10:54 | 0 |
| CSD | CSD/ISD | Mismatched | 11:28:46 - 11:39:33 | 77.5387 |
| CSD/ISD | CSD/ISD | Matched | 11:56:56 - 12:06:38 | 64.7124 |
| CSD | CSD/ISD | Mismatched | 12:28:43 - 12:44:20 | 67.7299 |
| CSD | CSD/ISD | Mismatched | 12:57:37 - 13:07:28 | 56.5504 |
| CSD | CSD/ISD | Mismatched | 13:28:14 - 13:43:16 | 66.4919 |
| ISD | CSD/ISD | Matched | 13:56:31 - 14:06:35 | 76 |
| CSD | CSD/ISD | Mismatched | 14:17:18 - 14:32:37 | 74.4877 |
| CSD | CSD/ISD | Mismatched | 14:43:43 - 14:59:54 | 75.75 |
| CSD | CSD/ISD | Mismatched | 15:08:46 - 15:24:55 | 72.4384 |
| CSD | CSD/ISD | Mismatched | 15:36:38 - 15:46:07 | 69.5397 |
| CSD | CSD/ISD | Mismatched | 15:54:29 - 16:06:31 | 76.6025 |
| CSD/ISD | CSD/ISD | Matched | 16:18:36 - 16:26:47 | 66.5 |
| CSD/ISD | CSD/ISD | Matched | 16:40:19 - 16:57:20 | 72.9878 |
| CSD | CSD/ISD | Mismatched | 17:13:14 - 17:33:05 | 80.5 |
| CSD/ISD | CSD/ISD | Matched | 17:36:28 - 17:52:06 | 63.3614 |
| CSD | CSD/ISD | Mismatched | 18:01:53 - 18:06:49 | 46.5 |
| CSD | No Event | False Positive | 18:24:48 - 18:41:19 | 65.5 |
